# Glutathione-S-transferase-pi (GST-pi) expression in renal cell carcinoma

**DOI:** 10.15586/jkcvhl.2015.22

**Published:** 2015-02-22

**Authors:** Christina Kaprilian, Maria Horti, Kosmas Kandilaris, Andreas Skolarikos, Nikolaos Trakas, Ioannis Kastriotis, Charalambos Deliveliotis

**Affiliations:** 1 Department of Pathology, Sismanoglio General Hospital, Sismanogliou 1, Marousi 15126, Athens, Greece; 2 Department of Clinical Chemistry Sismanoglio General Hospital of Attica; 3 2nd Department of Urology Athens Medical School, Sismanoglio Hospital, Sismanogliou 1, Marousi 15126, Athens, Greece.

## Abstract

Multidrug resistance correlates with unfavourable treatment outcomes in numerous cancers including renal cell carcinoma. The expression and clinical relevance of Glutathione-S-transferase-pi (GST-pi), a multidrug resistance factor, in kidney tumors remain controversial. We analyzed the expression of GST-pi in 60 formalin-fixed, paraffin-embedded renal cell carcinoma samples by immunohistochemistry and compared them with matched normal regions of the kidney. A significantly higher expression of GST-pi was observed in 87% of clear cell carcinoma and 50% of papillary subtypes. GST-pi expression did not correlate with tumor grade or patient survival. GST-pi is unlikely to be a prognostic factor for renal cell carcinoma. However, further studies with large number of samples are warranted to establish the role of GST-pi, if any, in intrinsic or acquired resistance of renal cell carcinoma to conventional treatments.

## Introduction

Despite the introduction of targeted therapies, metastatic renal cell carcinoma (RCC) continues to maintain its reputation as a treatment-resistant cancer. About 30% of the patients display intrinsic resistance to targeted therapy while the remaining 70% who initially respond will eventually acquire resistance between 6 and 11 months (1–4). A better understanding of molecules that influence drug resistance may shed light into the mechanisms of resistance of this deadly disease. In this regard, Glutathione-S-transferase-pi (GST-pi), has attracted some attention in the past. GSTs are a family of detoxification enzymes that protect cells from exogenous and endogenous electrophiles (5). There are eight mammalian GST isoforms (6). RCC cell lines that are resistant to adriamycin (7) and cisplatin (8) have a higher expression of GST-pi when compared with parental cell lines. Ferric nitrilotriacetate-induced renal tumors in rats are associated with a higher expression of GST-pi (9). However, some studies reported no significant correlation between GST activity and drug resistance (10). Baseline expression of GST-pi in human RCC samples has also been controversial. Some studies show that GST-pi is decreased in RCC (11, 12) while others show no difference (13, 14). On the contrary, some studies report a higher expression of GST-pi in RCC (15–17). A recent evidence-based meta-analysis concluded no association between GST-pi polymorphisms and the development of RCC (18). In this study, we examined the expression of GST-pi on primary renal cancer specimens to assess the value of this protein in RCC, any possible association of this marker to prognosis or other clinicopathologic parameters, and finally the importance of its expression in tumor aggressiveness.

## Material and Methods

### Ethics approval

Approval for this study was obtained from the Scientific Committee of our hospital (Sismanoglio General Hospital, Athens, Greece), Directors of the Departments, and the University of Athens, Greece. Informed consent was obtained from patients before the collection of the samples.

### Patient samples

Formalin-fixed, paraffin-embedded archival renal tumors from 60 patients along with matched morphologically normal regions of kidneys were used in this study. The tissue samples were obtained from patients who underwent nephrectomy for kidney cancer at the Department of Pathology (Sismanoglio General Hospital, Athens, Greece) between 1997 and 2001. Medical records showed that none of the patients received any pre-operative therapy. Tumor grade was established according to the World Health Organization (WHO) criteria and the clinical staging was conducted according to the TNM classification scheme.

### Immunohistochemistry

The expression of GST-pi was examined by routine immunohistochemistry technique. In brief, the blocks were cut into 5µm sections, mounted onto polylysine-coated slides, dewaxed in xylene, rehydrated in ethanol, treated with Trilogy solution (Code S3307; DAKO, Denmark) and heated in a microwave for 15 minutes. The sections were peroxidase-incubated for 10 minutes using 3% hydrogen peroxide and incubated for 20 minutes at room temperature with the GST-pi primary antibody (Thermo Scientific; Cat. No. RB-050-A1) at 1: 100 dilution. Then Envision/HRP (CodeK4010; DAKO, Denmark) was used for 20 minutes, followed by diaminobenzidine (DAB) for 5 minutes. The slides were counterstained with hematoxyline for 5 minutes, dehydrated in in ethanol, cleared in xylene and mounted in DePex mounting medium. Two pathologists independently reviewed the slides without the knowledge of former diagnosis. The intensity of GST-pi immunoreactivity was scored as negative (<10% cells with intense positive cytoplasmic and/or nuclear expression) or positive (>10% cells with intense positive cytoplasmic and/or nuclear expression) when compared with the matched morphologically normal regions. The distal tubular cells have been reported to have elevated concentrations of GST-pi [19]. Therefore we used the distal renal tubular cells as positive control. For negative controls, the primary antibody was omitted.

### Statistical analysis

The statistical program SPSS for Windows, version 13.0 (Statistical Package for the Social Sciences, Chicago, IL, USA) was used. Correlation of staining with baseline characteristics was performed using chi-square test and Fisher’s exact test. Life tables were estimated by Kaplan-Meier statistics and survival curves were compared using the Log-rank test. Survival was calculated from the day of surgery. All P values were two-sided and 5% was chosen as the level of statistical significance.

## Results

**Figure 1.**
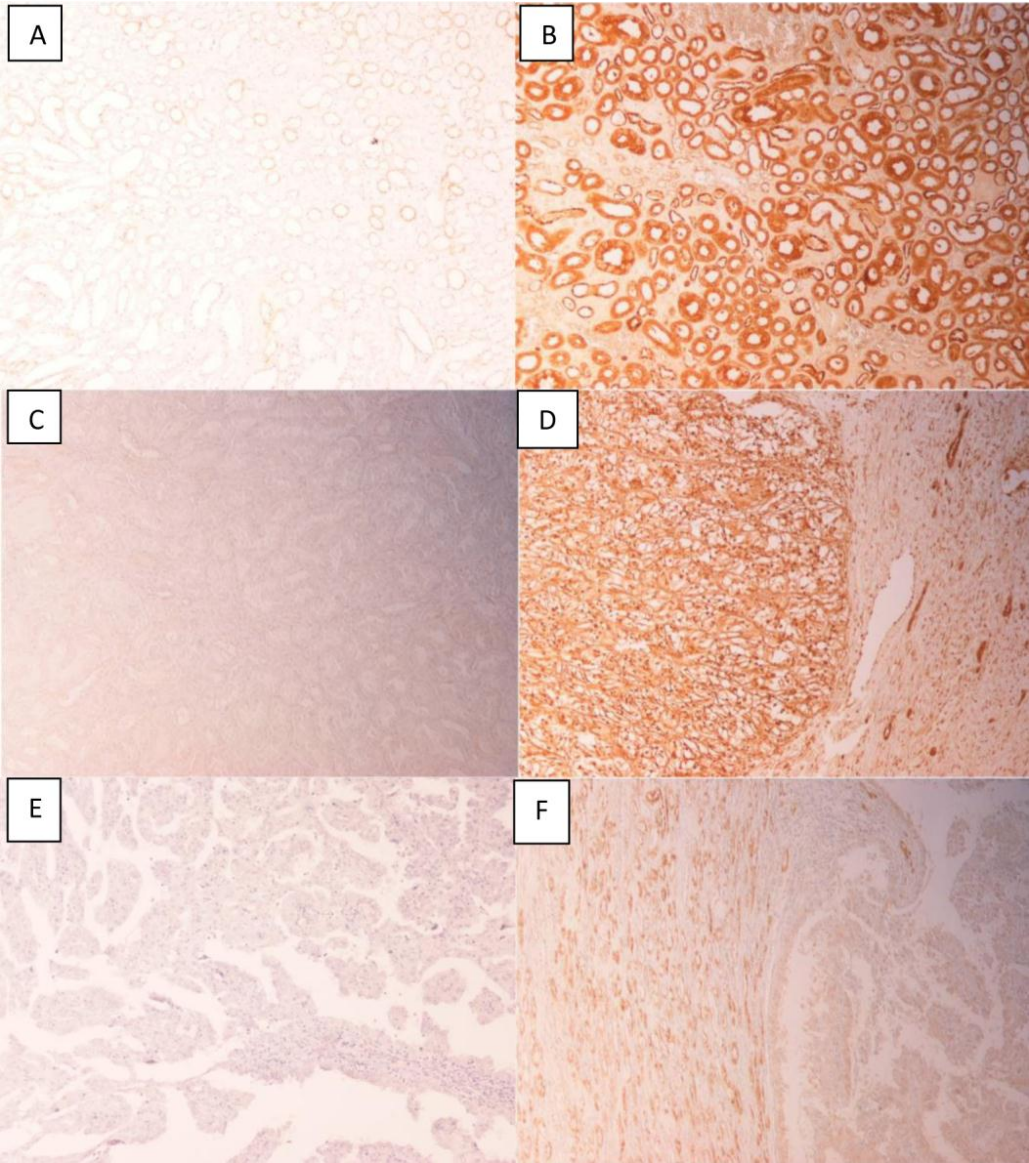
GST-pi expression in renal cell carcinoma. A, Normal distal tubular cells (negative control, primary antibody was excluded). B, Normal distal tubular cells acting as positive control for GST-pi expression. C, Normal cortical region showing weak or no expression of GST-pi. D, Clear cell RCC showing high expression of GST-pi. E, Papillary RCC showing weak expression of GST-pi. F, Papillary RCC showing high expression of GST-pi. Of the six papillary subtypes, 3 were positive and 3 were negative for GST-pi.

The characteristics, tumor grade and immunoreactivity of all 60 sample are shown in the **supplementary file**. Of the 60 samples, 54 were clear cell carcinomas and the remaining 6 were papillary subtypes. Eighty-seven percent (47/54) of clear cell and 50% (3/6) of papillary subtypes showed a higher expression of GST-pi when compared with matched normal regions. A representative photomicrograph for each subtype is shown in **Figure 1**. No significant correlation between GST-pi expression and tumor grade was observed **(Table 1)**. The median survival of GST-pi positive patients was 90.37 months, whereas for negative patients, it was 86.04 months. Given that only a small number of samples were negative, these results are insufficient to reach any reasonable conclusion on the effect of GST-pi on survival.

**Table 1.** Tumor grade and GST-pi expression

		**GST-pi Expression**		**Total**
		**Negative**	**Positive**	
Tumor grade	I-II	5	26(83.8%)	31
	III-IV	5	24(82.7%)	29
Total		10	50	60

## Discussion

RCC encompasses a set of tumors originating from the epithelial cells of the renal tubules (20). The two predominant subtypes of RCC are clear cell (60–70%) and papillary (10–15%). In our series, 54 (90%) cases were clear cell and 6 (10%) were papillary RCCs. GST-pi was increased in RCC, however, no correlation with tumor grade or stage was observed. Our findings contrast with previous studies that showed a decrease (11,12) or no change in GST-pi in RCC (13, 14). It has been suggested that a decrease in GST expression may represent a dedifferentiation program in RCC and that GST expression could be used as a prognostic marker in RCC (11). Our observations deviate from this assumption. Our findings are in agreement with studies that showed an increased expression of GST-pi in RCC. In one study 76% of samples were positive for GST-pi (21), and in another study, 100% of samples (12/12) were positive (17). The five-year survival for patients with GST-pi positive tumors was 88% versus 50% for those with GST-pi negative tumors (21), a pattern reflected in our findings. Both these studies employed immunohistochemistry to detect GST-pi expression. Toffoli and colleagues (16) analysed mRNA expression in 38 human RCC samples and showed a significantly higher GST-pi mRNA expression in RCC. In conclusion, an increase in expression of GST-pi was observed in our study. However it does not appear to have a correlation with tumor aggressiveness. GST-pi is unlikely to be a prognostic factor for RCC. Further studies, using a large number of samples, are warranted to establish the role of GST-pi in intrinsic or acquired resistance of RCC.
